# Organelle-anchored translation factories: the roles of neuronal RNA-binding protein condensates in organizing local protein synthesis in neurological diseases

**DOI:** 10.1093/procel/pwag026

**Published:** 2026-03-25

**Authors:** Semin Park, Hari Lim, Jin-A Lee

**Affiliations:** Department of Biological Sciences and Biotechnology, College of Life Sciences and Nanotechnology, Hannam University, Daejeon 34054, Republic of Korea; Department of Biological Sciences and Biotechnology, College of Life Sciences and Nanotechnology, Hannam University, Daejeon 34054, Republic of Korea; Department of Biological Sciences and Biotechnology, College of Life Sciences and Nanotechnology, Hannam University, Daejeon 34054, Republic of Korea

## Abstract

Neurons face a fundamental proteostasis challenge: synapses and axons located far from the soma must rapidly remodel their proteome during activity, stress, and development. While local protein synthesis has long been recognized as essential for meeting these demands, classical models largely focused on ribonucleoprotein granules as autonomous carriers of translationally silent mRNAs, treating membranous organelles as parallel logistics or metabolic systems. Recent work overturns this view, revealing that endosomes, lysosomes, axonal endoplasmic reticulum, mitochondria, and their contact sites actively function as mobile translation platforms. In this review, we propose an RBP-centered framework in which phase-separated condensates physically tether specific mRNA cohorts to organelle surfaces, coupling mRNA transport, translational control, and organelle dynamics into a unified network. By organizing recent discoveries into functional modules—long-range transport, localized translation, and stress buffering—this neuron-focused framework identifies organelle-anchored translation factories as a unifying principle of synaptic proteostasis and a broadly applicable design paradigm for highly polarized cells.

**Keywords** RNA-binding proteins, ribonucleoprotein granules, neuronal local translation, organelle-anchored translation

## Introduction

Neurons face an extreme proteostasis challenge: synapses and axons located far from the soma must remodel their proteome within seconds to minutes in response to activity, development, and stress. These spatial and temporal demands cannot be satisfied by slow, capacity-limited soma-to-synapse protein transport alone (Daskin et al., 2025; Harbauer et al., 2022; Maday et al., 2014; Rajgor et al., 2021). Local mRNA translation therefore provides an essential solution, enabling on-site synthesis of synaptic, metabolic, and proteostasis factors from transported mRNAs (Alecki et al., 2024; Shigeoka et al., 2016; Swanger and Bassell, 2013).

For decades, dominant frameworks emphasized ribonucleoprotein (RNP) granules and RNA-binding proteins (RBPs) as autonomous carriers of translationally repressed mRNAs. These complexes were thought to deliver transcripts to distal compartments, where translation would later be activated by local signals (Glock et al., 2017; Holt and Schuman, 2013; Jung et al., 2012; Kiebler and Bassell, 2006). In parallel, membranous organelles—including endosomes, lysosomes, axonal endoplasmic reticulum (ER), mitochondria, and their contact sites—were largely viewed as independent trafficking, metabolic, or degradative systems, shaping synaptic function indirectly through cargo transport, energy supply, or turnover (Lee et al., 2018; Maday et al., 2014; Yperman and Kuijpers, 2023; Zhang et al., 2022).

Over the past decade, this conceptual separation has been increasingly challenged. A growing body of work demonstrates that membranous organelles can function as spatially addressable translation platforms, rather than passive support structures. RBPs—often acting as phase-separating adaptors—physically tether selected mRNA cohorts to organelle surfaces, thereby coordinating long-range transport, translational control, and organelle dynamics (Bauer and Koppers, 2025; Fernandopulle et al., 2021; Vargas et al., 2022).

Representative examples illustrate this emerging model, in which RNP condensates are tethered to specific organelles—including lysosomes, endosomes, axonal ER tubules, and mitochondria—through defined adaptor complexes (e.g., ANXA11, Rab7, FERRY, p180/RRBP1, and SYNJ2BP–SYNJ2) to enable long-range transport and spatially restricted translation in distal axons (Cioni et al., 2019; Hoffmann et al., 2023; Koppers et al., 2024; Liao et al., 2019; Schuhmacher et al., 2023; Vargas et al., 2022). Collectively, these studies indicate that neuronal local translation is frequently executed by discrete condensate–membrane units, rather than by diffusely distributed cytosolic polysomes, with translational output governed by organelle identity, motility, and contact behavior (Glock et al., 2017; Shigeoka et al., 2016; Spillane et al., 2013).

Despite this rapid progress, existing reviews have notable limitations. They are often organized organelle-by-organelle or focus selectively on either RNP condensates or membrane contact sites. As a result, it remains unclear how these mechanisms integrate into a unified strategy for synaptic proteostasis, or how selective disruption of specific organelle–RBP interfaces contributes to neurodegeneration (Bauer and Koppers, 2025; Klein et al., 2016; Otis and Mowry, 2023; Vargas et al., 2022).

Here, we advance an RBP-centered framework in which phase-separated condensates tethered to lysosomes, endosomes, ER tubules, mitochondria, mitochondria–ER contact sites (MERCs), and synapses function as organelle-anchored translation factories. These assemblies route, protect, and translate neuronal mRNAs, linking subcellular positioning to translational competence. We propose that such factories represent an extreme but instructive realization of spatial translation strategies that operate more broadly in polarized cells (Bethune et al., 2019; Pushpalatha and Besse, 2019; Vargas et al., 2022). Within this framework, RBP-containing RNP assemblies, including phase-separated condensates in some contexts, may act as organizing platforms that couple mRNA transport, translational control, and organelle dynamics, while leaving room for additional mechanisms. Throughout this review, we use “RBP” to refer to individual RNA-binding proteins and “RNP” to refer to RNA–protein assemblies that may include mRNAs, RBPs, ribosomes, and associated cofactors.

This review first outlines shared biophysical principles—multivalency, phase separation, and regulated tethering—that enable RBPs to assemble membrane-proximal condensates and dynamically switch translation on or off in response to activity and stress (Banani et al., 2017; Holt et al., 2019; Mittag and Parker, 2018; Vargas et al., 2022). It then synthesizes recent findings by functional roles within this distributed network, encompassing long-range transport modules, local translation hubs, and stress-buffering nodes. By emphasizing common design logic across organelles, the review highlights mechanistic links to disorders such as amyotrophic lateral sclerosis and Charcot–Marie–Tooth disease (Bauer and Koppers, 2025; Cioni et al., 2019; Fernandopulle et al., 2021; Liao et al., 2019).

Finally, the review discusses open questions and experimental strategies, including efforts to decode the “tethering code,” visualize condensate–organelle dynamics *in vivo*, and exploit organelle-coupled translation principles to stabilize local proteostasis in vulnerable circuits (Bauer and Koppers, 2025; Shigeoka et al., 2016; Vargas et al., 2022). This perspective predicts that disrupting distinct organelle–RBP tethers will produce compartment-specific translational defects, even when global RNA granule dynamics appear intact, thereby offering a testable framework for future studies (Alecki et al., 2024; Glock et al., 2017; Spillane et al., 2013).

## Local translation and the synaptic proteostasis challenge

### Soma-to-synapse transport limits in extended neurons

Neurons must maintain proteostasis in axons and dendrites that can extend hundreds of micrometers or more from the soma, while synapses remodel their proteome within seconds to minutes during plasticity and stress responses (Alecki et al., 2024; Daskin et al., 2025). Slow, capacity‑limited anterograde transport of newly synthesized proteins—typically millimeters per day for cytosolic cargo—cannot by itself meet these rapid, input‑specific demands at distal synapses, especially when many protein species must be delivered simultaneously (Maday et al., 2014; Roy, 2014).

Local protein synthesis within dendrites and axons therefore provides a necessary complement, enabling on‑site production of synaptic, metabolic, and chaperone proteins that maintain compartment‑specific proteostasis at baseline and after neuronal activity or proteotoxic stress (Alecki et al., 2024; Daskin et al., 2025; Swanger and Bassell, 2013). However, the extreme spatial dispersion of synapses also imposes organizational constraints: locally translated proteins must be produced at the right place, time, and quantity, rather than diffusely throughout the cytosol.

High‑resolution imaging and metabolic labeling now show that ribosomes and nascent proteins are present at most synapses, and that locally synthesized protein levels scale with synaptic activity and plasticity induction, underscoring how local translation directly buffers soma‑to‑synapse transport limits (Hafner et al., 2019; Schuhmacher et al., 2023). These observations imply that local translation is not stochastic or uniformly distributed, but instead relies on spatial coordination mechanisms that concentrate translational machinery at functionally relevant subcellular sites, setting the stage for the organelle‑anchored translation platforms discussed below.

### Classical models of RNP granules and local translation

Early models of neuronal RNA localization posited that mRNAs are packaged into ribonucleoprotein (RNP) granules, where translationally silenced transcripts are escorted from the soma along microtubules into dendrites and axons (Glock et al., 2017; Jung et al., 2012; Kiebler and Bassell, 2006). These granules were described as dynamic, membraneless assemblies enriched in RNA‑binding proteins (RBPs), translation factors, and regulatory RNAs that together determine which mRNAs are transported, how long they remain repressed, and when they are released for translation (Fig. 1A) (Glock et al., 2017; Kiebler and Bassell, 2006).

**Figure 1.**
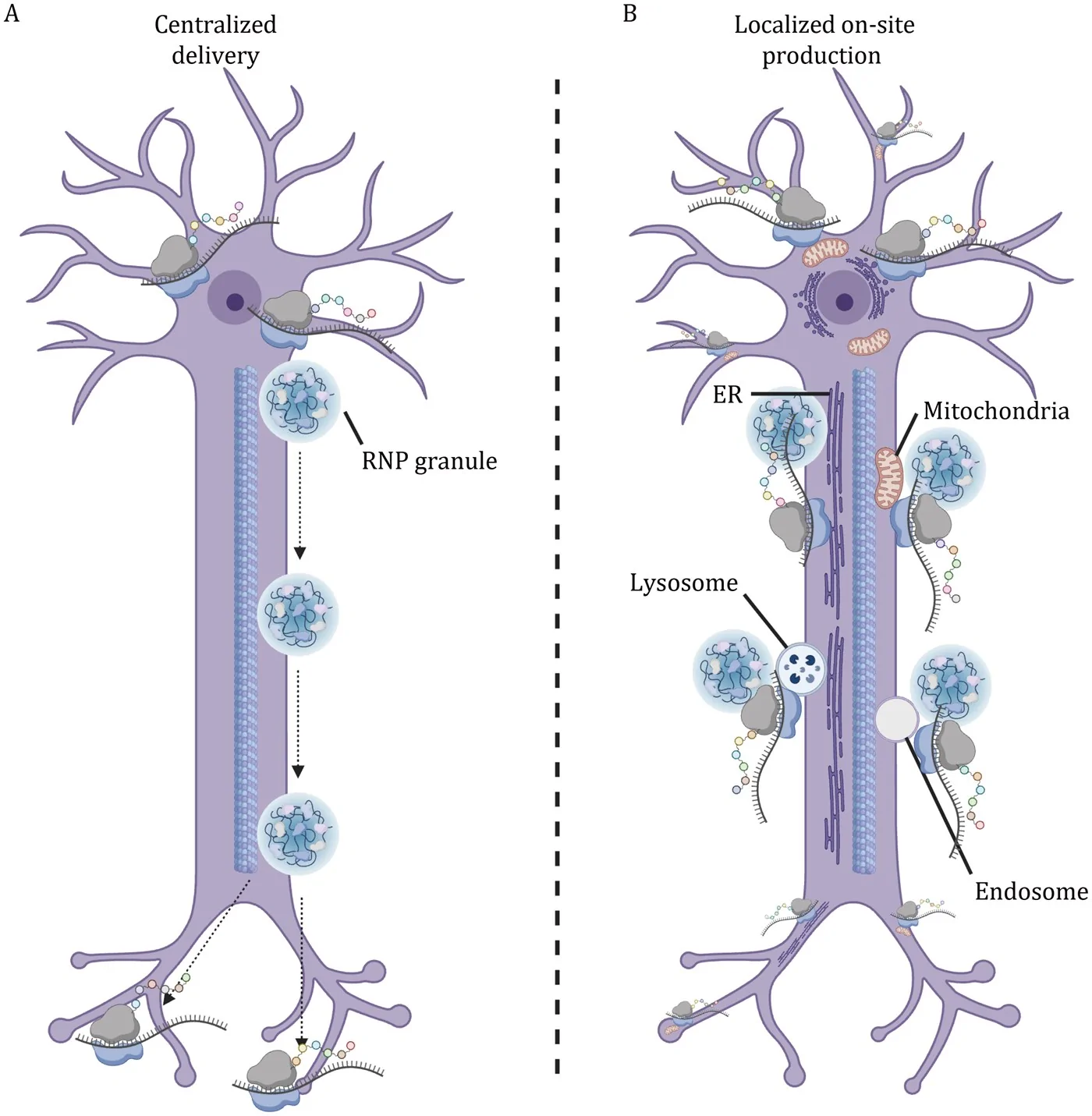
**Conceptual comparison between the classical view of neuronal mRNA transport and emerging models of organelle-anchored local translation**. (A) Classical model. Messenger RNAs are packaged into translationally repressed ribonucleoprotein (RNP) granules in the soma and transported bidirectionally along microtubules into axons and dendrites. Upon reaching distal sites such as synapses, RNP granules are thought to disassemble, allowing ribosomes to engage mRNAs and initiate translation in the surrounding cytosol. In this model, membranous organelles primarily serve supportive or metabolic roles and are not explicitly coupled to translational control. (B) Organelle-anchored translation factory model. In contrast, accumulating evidence suggests that local translation is spatially organized on membranous organelles, including the endoplasmic reticulum (ER), mitochondria, lysosomes, and endosomes. These organelles recruit RNA-binding protein (RBP) condensates and associated translational machinery, forming mobile or stationary translation platforms that operate throughout neuronal compartments, including dendrites and axons. Organelle contacts provide positional cues, metabolic support, and regulatory signals that coordinate mRNA activation, translational output, and proteostasis in a compartment-specific manner. This framework redefines local translation as an organelle-coupled process rather than a purely cytosolic event.

In this classical framework, synaptic activity, neuromodulators, or guidance cues remodel RNP granules—via signaling to RBPs and local changes in phosphorylation or RNA content—allowing selected mRNAs to disengage from repressive complexes and initiate translation near activated synapses (Holt and Schuman, 2013; Jung et al., 2012; Schuhmacher et al., 2023). These models successfully explained input‑specific translation and synapse‑restricted protein synthesis and established RNP granules as central regulators of neuronal mRNA fate.

For many years, however, they treated membrane‑bound organelles largely as a separate logistical layer, with local translation envisioned as occurring on free cytosolic polysomes positioned near synapses but not obligatorily coupled to specific organelles (Jung et al., 2012; Kiebler and Bassell, 2006). As a result, classical frameworks offered limited mechanistic insight into how translationally competent mRNAs are spatially organized, stabilized, or coordinated with local energy supply, membrane trafficking, and stress responses in extended neuronal processes. This left unresolved how local translation is physically anchored, dynamically repositioned, or selectively preserved under transport stress or global translational repression, issues that became clearer with the discovery of organelle‑associated translation platforms (Fig. 1B).

## Dynamic organelle networks in axons and dendrites

### Endosomes and lysosomes as neuronal logistics hubs

Endosomes and lysosomes form highly dynamic trafficking networks in axons and dendrites, supporting receptor recycling, cargo sorting, and degradation while continuously moving between the soma and distal synapses (Farias et al., 2017; Lasiecka and Winckler, 2016; Maday et al., 2014). Early endosomes integrate endocytic cargo and signaling receptors, then mature into Rab7‑positive late endosomes that retrogradely transport neurotrophic signals and anterogradely deliver membrane components to pre‑ and postsynaptic sites (Maday et al., 2014; Millecamps and Julien, 2013).

Lysosomes and lysosome‑related organelles, distributed throughout axons and dendrites, mediate local degradation of membrane proteins and damaged organelles, preserving synaptic composition and preventing toxic cargo accumulation far from the soma (Farias et al., 2017; Lasiecka and Winckler, 2016). Collectively, these endolysosomal compartments were historically viewed as logistics hubs specialized for sorting, signaling, and degradation, rather than as direct sites of mRNA transport or protein synthesis (Lasiecka and Winckler, 2016; Maday et al., 2014).

This view implicitly separated membrane trafficking from local translational control and suggested that endosomes and lysosomes influence synaptic proteostasis only indirectly through cargo delivery or degradation. However, their pronounced motility, spatial reach, and regulated pausing in axons and dendrites make them well suited to couple long‑range mRNA transport with localized protein synthesis, as highlighted by recent work on Rab7‑positive endosome‑coupled translation in axons.

This realization reframes endosomes and lysosomes not merely as carriers or degradative endpoints but as dynamic scaffolds that recruit RNA‑binding proteins, ribosomes, and translationally poised mRNAs, setting the stage for their emerging roles as mobile translation platforms.

### Axonal ER, mitochondria, and organelle contact sites

Axons and dendrites contain an extensive, largely tubular endoplasmic reticulum (ER) network that is continuous with somatic ER yet structurally distinct, supporting local lipid synthesis, calcium signaling, and membrane protein trafficking along neurites (Wu et al., 2017; Yperman and Kuijpers, 2023). Mitochondria are strategically positioned at sites of high energy demand, including presynaptic boutons and dendritic spines, where they produce ATP and buffer calcium to sustain neurotransmission and plasticity (Devine and Kittler, 2018; Lee et al., 2018). These organelles form close associations at mitochondria–ER contact sites (MERCs), which facilitate lipid exchange, calcium transfer, and coordination of metabolic signaling across compartments (Rowland and Voeltz, 2012; Paillusson et al., 2016). Before the recent shift toward organelle‑coupled translation, this ER–mitochondria network in axons and dendrites was viewed mainly as a scaffold for trafficking and metabolic homeostasis, with proteostatic roles confined to folding and quality control within the ER lumen or mitochondrial matrix rather than direct participation in local mRNA handling (Rowland and Voeltz, 2012; Wu et al., 2017).

This perspective implicitly separated metabolic support from translational regulation, assuming that local protein synthesis occurs independently of the organelles that supply energy, lipids, and calcium at synapses. However, the intimate spatial coupling of ER tubules, mitochondria, and MERCs within axons and dendrites positions these structures as ideal platforms for coordinating mRNA localization, translational control, and metabolic state.

Rather than serving solely as passive resource suppliers, the axonal ER–mitochondria network provides a physical and signaling framework in which RBPs, ribosomes, and translationally poised mRNAs can be locally anchored, regulated, and selectively activated in response to neuronal activity or stress. This conceptual shift motivates viewing ER tubules, mitochondria, and their contact sites as active decision nodes that integrate bioenergetic cues with spatially precise local translation throughout axons and dendrites.

## Organelles as mobile translation platforms

### RBP-mediated tethering and phase-separated condensates

Recent work shows that many RNA‑binding proteins (RBPs) that control local translation contain low‑complexity or intrinsically disordered regions that undergo liquid–liquid phase separation, forming condensates that concentrate specific mRNAs, ribosomes, and regulatory factors at defined subcellular sites (Banani et al., 2017; Mittag and Parker, 2018). These condensates locally enrich translational components, creating discrete biochemical microenvironments within the crowded neuronal cytoplasm.

When these RBPs also contain membrane‑binding domains or interact with organelle‑resident adaptors, their condensates can dock onto lysosomes, endosomes, ER tubules, mitochondria, or synaptic vesicle clusters, forming organelle‑anchored translation hubs rather than freely diffusing cytosolic granules (Bauer and Koppers, 2025; Vargas et al., 2022). Membrane tethering transforms phase‑separated condensates from transient RNA assemblies into spatially fixed, regulatable translation factories whose position is defined by organelle identity and dynamics.

Such tethered condensates create multivalent platforms that both shield mRNAs from untimely translation and position them for rapid activation when local signals—such as calcium influx, kinase activity, or metabolic stress—alter RBP phase behavior and modulate recruitment of translation machinery (Holt et al., 2019; Naskar et al., 2023; Vargas et al., 2022). In this way, RBPs function not only as molecular switches of translation but also as architectural linkers that couple RNA fate decisions to membrane traffic, organelle positioning, and local metabolic state in neurons, in a design that could be reused in other polarized cell types.

### ANXA11-dependent lysosomal hitchhiking

A prominent example of organelle‑anchored condensates is Annexin A11 (ANXA11), which uses an N‑terminal low‑complexity domain to form RNP‑rich condensates and a C‑terminal annexin‑repeat domain to bind acidic phospholipids on lysosomal membranes in a calcium‑dependent manner (Liao et al., 2019; Nixon-Abell et al., 2025). This dual architecture enables ANXA11 to act both as a phase‑separating RNP binder and as a membrane tether, directly linking condensates to motile lysosomal surfaces (Liao et al., 2019).

In axons, this organization allows RNP granules to “hitchhike” on lysosome‑related organelles, coupling long‑range lysosomal transport to the delivery of translationally competent mRNAs at distal sites (Fig. 2A) (Liao et al., 2019; Vargas et al., 2022). Lysosomal motility thereby gives RNPs access to regions far beyond the reach of passive diffusion, positioning ANXA11‑tethered condensates as active vehicles for spatially precise RNA logistics (Liao et al., 2019).

**Figure 2.**
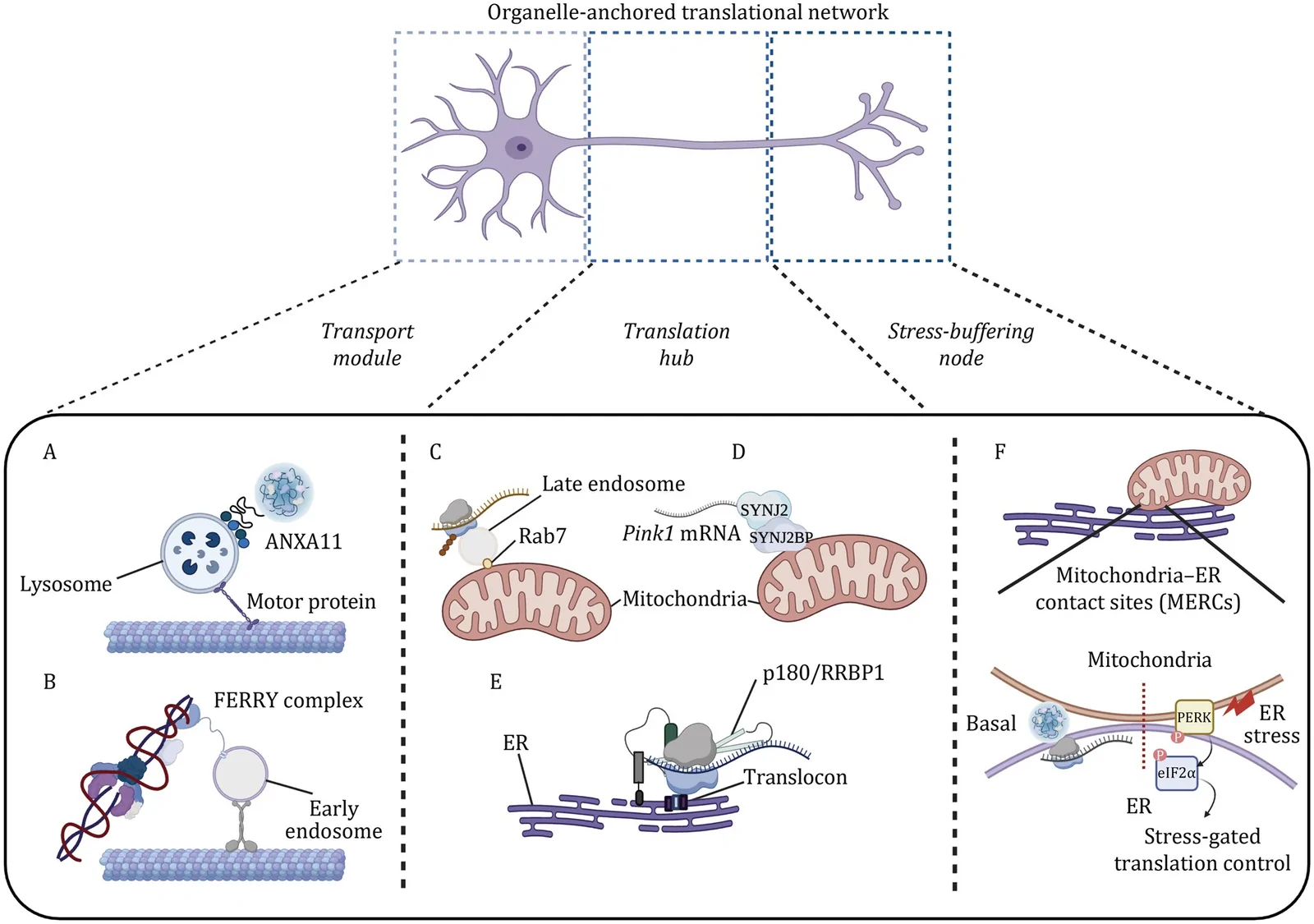
**Organelle-anchored translational networks coordinate neuronal local protein synthesis**. RNA-binding protein (RBP) condensates associate with distinct membranous organelles to form a distributed translational network that supports spatially regulated protein synthesis across neuronal compartments. (A–B) Transport module. Translationally repressed RBP condensates are coupled to motile organelles to enable long-range mRNA delivery. (A) ANXA11-mediated tethering of RNP granules to lysosomes allows microtubule-dependent transport into distal neuronal processes. (B) The FERRY complex recruits selected mRNAs to early endosomes, providing an additional carrier system for directed mRNA trafficking. (C–E) Translation hub. Local translation is selectively activated at organelle-associated platforms. (C) Rab7-positive late endosomes pause at mitochondria, creating sites where localized translation can be coordinated with mitochondrial function. (D) The SYNJ2BP–SYNJ2 complex anchors specific mRNAs, such as Pink1, to the mitochondrial surface, enabling compartment-specific translation. (E) p180/RRBP1-enriched endoplasmic reticulum (ER) microdomains organize ribosome engagement and translocon-associated translation, supporting efficient local protein production. (F) Stress-buffering node. Mitochondria–ER contact sites (MERCs) function as stress-responsive translational nodes. Under basal conditions, translation proceeds normally, whereas during ER stress, signaling pathways such as PERK–eIF2α modulate local translational output, preserving the synthesis of critical proteins while globally restraining translation.

Disrupting ANXA11–lysosome tethering—by mutating either the low‑complexity region or ALS‑linked residues within the annexin core—impairs RNP motility, causes aberrant clustering of both RNPs and lysosomes, and compromises axonal integrity (Liao et al., 2019; Marchica et al., 2025). These phenotypes show how failure of a single RBP‑mediated tether can jointly perturb mRNA transport, local translation, and organelle dynamics, providing a mechanistic bridge between defective RNA logistics and axonal degeneration in ALS (Liao et al., 2019; Marchica et al., 2025). ANXA11‑dependent lysosomal hitchhiking thus offers a paradigmatic example of how organelle‑anchored condensates implement mobile translation platforms in neurons.

### Rab7-positive late endosomes as axonal translation sites

Rab7‑positive late endosomes in axons act not only as retrograde signaling carriers but also as platforms that associate with ribosomes and mRNAs, forming mobile translation sites that pause near mitochondria to synthesize proteins required for mitochondrial maintenance and axonal health (Fig. 2C) (Cioni et al., 2019; Vargas et al., 2022). This dual role places late endosomes at the interface between long‑range trafficking and local proteome control (Cioni et al., 2019).

Live‑imaging and ribopuromycylation assays show that these late endosomes locally translate nuclear‑encoded mitochondrial transcripts and other axonal mRNAs while stationed at mitochondria, directly coupling endosomal trafficking to organelle support and energy homeostasis (Cioni et al., 2019; Vargas et al., 2022). The regulated pausing of Rab7‑positive endosomes at mitochondria thus functions as a spatial checkpoint where mRNA localization, translational activation, and organelle demand are integrated (Cioni et al., 2019).

Disease‑linked RAB7A mutations that alter late endosome dynamics reduce endosome‑associated translation and compromise mitochondrial function in axons, suggesting that defects in this endosomal translation axis contribute to the vulnerability seen in Charcot–Marie–Tooth disease type 2B and related neuropathies (Cioni et al., 2019; Millecamps and Julien, 2013). These findings indicate that impairing endosomal positioning or dynamics can selectively disrupt local translation without globally affecting RNA granule formation, reinforcing the idea of organelle‑specific translational failure modes in neurodegenerative disease (Bauer and Koppers, 2025; Cioni et al., 2019).

### Early endosomal FERRY complexes and mRNA recruitment

Earlier in the endocytic pathway, Rab5‑positive early endosomes recruit mRNAs and ribosomes via the FERRY complex, a pentameric Rab5 effector that directly binds selected transcripts and links them to early endosomal membranes (Quentin et al., 2023; Riffe and Downes, 2025; Schuhmacher et al., 2023). Through this RNA‑binding activity, FERRY gives early endosomes the capacity to act as selective entry points for mRNAs into the organelle‑coupled translation network (Fig. 2B) (Quentin et al., 2023; Schuhmacher et al., 2023).

Proteomic and RNA‑binding studies show that FERRY recognizes subsets of mRNAs, including those encoding mitochondrial and metabolic proteins, positioning early endosomes as RNA‑recruiting, transport‑competent carriers that can hand off transcripts to downstream compartments or local translation sites (Bauer and Koppers, 2025; Schuhmacher et al., 2023). This selectivity suggests that early endosomes participate in an initial sorting step that biases mRNA fate toward organelle‑associated translation rather than diffuse cytosolic distribution (Quentin et al., 2023; Schuhmacher et al., 2023).

This early endosome–FERRY axis broadens endosomal involvement in RNA logistics from late endosome‑based translation to a multi‑step pathway in which distinct endosomal stages sequentially capture, transport, and ultimately support translation of neuronal mRNAs (Schuhmacher et al., 2023; Vargas et al., 2022). Together with Rab7‑positive late endosomes, FERRY‑decorated early endosomes define a hierarchical endosomal pipeline that couples mRNA selection to transport dynamics and translational activation in axons, and emerging genetic data link FERRY mutations to neurodevelopmental and neurodegenerative phenotypes, underscoring the physiological importance of this axis.

### Axonal ER tubules and p180/RRBP1 translation microdomains

Contrary to the long‑held view that rough ER and most co‑translational protein synthesis are restricted to the soma, thin ER tubules extend throughout axons and can host local translation (Koppers et al., 2024; Wu et al., 2017; Yperman and Kuijpers, 2023). These axonal ER tubules form a continuous but spatially patterned membrane network that supports compartmentalized protein synthesis far from the soma (Wu et al., 2017; Yperman and Kuijpers, 2023).

In mammalian neurons, the ribosome receptor p180/RRBP1 is distributed along axonal ER and forms discrete microdomains where ribosomes and mRNAs are concentrated, creating nanoscale translation sites for membrane and secreted proteins needed for axon growth and synapse development (Koppers et al., 2024). By tethering ribosomes to ER membranes, p180/RRBP1 converts axonal ER tubules into spatially fixed translation platforms that resemble membrane‑anchored condensates rather than diffuse cytosolic polysomes (Fig. 2E) (Koppers et al., 2024; Wu et al., 2017).

Disrupting p180/RRBP1‑mediated ribosome tethering reduces local protein synthesis, alters axon morphology, and impairs synapse formation, emphasizing that axonal ER tubules are bona fide translation platforms rather than passive conduits of somatic cargo (Koppers et al., 2024; Yperman and Kuijpers, 2023). These findings indicate that ER‑associated translation microdomains are a critical node in the organelle‑coupled translation network, linking membrane trafficking, local proteome remodeling, and neuronal circuit assembly (Koppers et al., 2024; Wu et al., 2017).

### SYNJ2BP-SYNJ2-guided translation on motile mitochondria

The outer mitochondrial membrane protein SYNJ2BP functions as an RNA‑binding adaptor that anchors specific nuclear‑encoded mitochondrial mRNAs at mitochondria, supporting local translation during stress recovery (Harbauer et al., 2022; Qin et al., 2021). By directly coupling mRNA binding to the mitochondrial surface, SYNJ2BP establishes mitochondria as spatially defined sites of mRNA retention and translation rather than passive recipients of newly synthesized proteins (Harbauer et al., 2022; Qin et al., 2021).

In neurons, SYNJ2BP cooperates with the neuronal splice isoform of synaptojanin‑2 (SYNJ2) to co‑transport Pink1 mRNA on motile mitochondria and promote its translation when mitochondria pause in distal axons, linking mitochondrial motility to local production of short‑lived PINK1 required for mitophagy and quality control (Fig. 2D) (Harbauer et al., 2022; Qin et al., 2021; Vargas et al., 2022). This mechanism ensures that translational output is temporally and spatially matched to local organelle demand (Harbauer et al., 2022).

The SYNJ2BP–SYNJ2 axis is modulated by signaling pathways such as insulin and AMPK, connecting metabolic state to mitochondrial mRNA anchoring and highlighting mitochondria as regulated translation hubs within the broader organelle‑coupled RNA logistics network (Alecki et al., 2024; Hees et al., 2024; Vargas et al., 2022). Together, these findings depict mitochondria not merely as consumers of locally synthesized proteins but as active organizers of mRNA localization and translation whose dynamics directly shape neuronal proteostasis.

### MERCs as stress-responsive translation “safe havens”

Mitochondria–ER contact sites (MERCs) are specialized interfaces that coordinate lipid exchange, calcium signaling, and stress‑responsive pathways in neurons (Paillusson et al., 2016; Rowland and Voeltz, 2012; Watanabe and Yamanaka, 2025). Positioned between two organelles central to proteostasis and metabolic homeostasis, they are well suited to modulate local translational states during cellular stress (Kohler and Kohler, 2024; Paillusson et al., 2016). Emerging evidence suggests that MERCs act not as constitutive translation sites but as regulatory microdomains that locally shape translational outcomes in response to stress signals (Bassot et al., 2021; Watanabe and Yamanaka, 2025).

During ER stress and integrated stress response (ISR) activation, PERK and associated signaling components accumulate at ER–mitochondria interfaces, where they can locally regulate eIF2α phosphorylation and downstream translational control (Fig. 2F) (Costa-Mattioli and Walter, 2020; Lebeau et al., 2018; Verfaillie et al., 2012). While global PERK activation suppresses bulk cytosolic translation, compartmentalized signaling at MERCs may permit selective maintenance or rapid recovery of translation for subsets of mitochondrial and stress‑adaptive proteins near these contact sites (Costa-Mattioli and Walter, 2020; Lebeau et al., 2018; Verfaillie et al., 2012). Direct visualization of nascent peptide synthesis at MERCs is still limited, so current models emphasize spatial regulation of translational competence rather than continuous local translation (Brar et al., 2024; Costa-Mattioli and Walter, 2020).

In neurons, disrupting MERC architecture or PERK‑dependent signaling at these junctions impairs mitochondrial calcium handling, lipid homeostasis, and stress resilience, indirectly affecting local proteostasis in axons and synapses (Paillusson et al., 2016; Ronayne and Latorre-Muro, 2024; Verfaillie et al., 2012). These data support a model in which MERCs bias translational priorities under stress by modulating when and where translational repression is imposed or lifted, rather than operating as autonomous translation factories (Brar et al., 2024). From this perspective, MERCs act as stress‑gated decision nodes that couple organelle crosstalk to localized translational control, helping neurons prioritize mitochondrial maintenance and synaptic stability during proteotoxic or metabolic challenges (Casas-Martinez et al., 2024; Paillusson et al., 2016; Perea et al., 2023; Ronayne and Latorre-Muro, 2024).

Together, current evidence positions MERCs as conditional regulators within the broader organelle‑anchored translation network—interfaces that tune translational permissiveness in space and time rather than constitutively hosting protein synthesis (Ronayne and Latorre-Muro, 2024). Defining the extent to which MERCs directly support local translation, and identifying the RBPs and signaling complexes that enforce this control, remains an important challenge for future work (Costa-Mattioli and Walter, 2020; Ronayne and Latorre-Muro, 2024).

## An integrated network of organelle-anchored translation factories

### From organelle-by-organelle views to a logistics-and-production network

Early studies described lysosomes, endosomes, ER, mitochondria, and synaptic vesicle clusters separately, emphasizing their individual roles in trafficking, degradation, membrane protein biogenesis, or energy production (Lee et al., 2018; Maday et al., 2014; Vargas et al., 2022; Yperman and Kuijpers, 2023). This compartmentalized view yielded key mechanistic insights but implicitly treated each organelle as an isolated station, leaving unresolved how local translation is coordinated across extended neuronal processes (Fig. 2).

The realization that RBPs tether RNP condensates to these organelles—via ANXA11 on lysosomes, Rab7 platforms on late endosomes, FERRY on early endosomes, p180/RRBP1 on axonal ER, SYNJ2BP on mitochondria, and PERK complexes at MERCs—has reframed them as nodes in a distributed logistics‑and‑production network for local translation rather than as standalone units (Bauer and Koppers, 2025; Cioni et al., 2019; Koppers et al., 2024; Liao et al., 2019; Schuhmacher et al., 2023; Vargas et al., 2022). In this integrated framework, organelles and condensates jointly determine where mRNAs travel, pause, and are decoded, enabling neurons to orchestrate proteome remodeling across long processes with sub‑synaptic precision (Bauer and Koppers, 2025; Daskin et al., 2025).

This network perspective further emphasizes that local translation is an emergent property of organelle coordination rather than a mere sum of independent translational events, offering a conceptual basis for how neurons achieve speed, specificity, and robustness in proteostasis.

### Functional roles: transport vehicles, translation hubs, and stress buffers

Different organelle–RBP assemblies in this network specialize in complementary functions. Lysosomes and specific late endosomes act as long‑range transport vehicles that ferry RNPs into axons and dendrites, whereas Rab7 platforms, axonal ER microdomains, and mitochondria serve as translation hubs where ribosomes and selected mRNAs are locally concentrated and activated (Cioni et al., 2019; Hung et al., 2017; Koppers et al., 2024; Liao et al., 2019).

This division of labor allows neurons to decouple mRNA transport from translational activation, so transcripts can be carried over long distances while remaining silenced until they reach the correct subcellular sites (Cioni et al., 2019; Liao et al., 2019). MERCs and related contact sites function as stress buffers that maintain translation of compartment‑critical proteins under global repression, preserving mitochondrial function and synaptic resilience during proteotoxic or metabolic challenge (Costa-Mattioli and Walter, 2020; Hetz and Saxena, 2017; Paillusson et al., 2016).

By selectively sustaining local translation when bulk protein synthesis is curtailed, these contacts impose a hierarchy on which compartments retain translational capacity within a neuron. Together, these roles define a modular architecture in which transport, synthesis, and stress adaptation are physically coupled yet flexibly reconfigurable, offering a mechanistic explanation for how neurons combine speed, specificity, and robustness in local proteostasis (Bauer and Koppers, 2025; Vargas et al., 2022).

The modularity of this network further implies that individual organelle–RBP assemblies can be selectively tuned or disrupted, generating compartment‑specific translational phenotypes without globally disturbing RNA metabolism (Bauer and Koppers, 2025; Daskin et al., 2025).

### Biophysical principles: multivalency, phase separation, and regulated tethering

At the molecular level, integration of organelles with local translation relies on multivalent RBPs whose low‑complexity domains drive phase separation, generating condensates that enrich mRNAs and translational machinery far above cytosolic levels (Banani et al., 2017; Mittag and Parker, 2018). This enrichment offers a biophysical solution for rapid, localized translation within the dilute, crowded environment of neuronal processes.

Coupling these condensates to organelle membranes—via lipid‑binding motifs, transmembrane segments, or adaptors such as ANXA11, FERRY, SYNJ2BP, and p180—anchors droplets in space while leaving their material properties tunable by post‑translational modifications and signaling (Hung et al., 2017; Koppers et al., 2024; Liao et al., 2019; Schuhmacher et al., 2023). Regulated tethering thus converts otherwise dynamic condensates into spatially addressable translation units, allowing neurons to assign distinct translational tasks to specific organelles without hard‑wiring them into static structures (Bauer and Koppers, 2025; Vargas et al., 2022).

Activity‑ and stress‑dependent control of condensate assembly, dissolution, and composition then acts as a molecular switch that links neuronal firing, calcium influx, or unfolded‑protein responses to rapid changes in which mRNAs are transported, stored, or translated at each organellar site (Bauer and Koppers, 2025; Holt et al., 2019; Vargas et al., 2022). Together, multivalency, phase separation, and regulated tethering define design principles that give neuronal translation networks both stability and adaptability and may provide a general blueprint for organelle‑proximal translation across cell types.

## Disease mechanisms at organelle-RBP interfaces

To facilitate a disease-oriented overview for readers, we summarize representative RNP-associated components implicated in neuronal local translation and neurological disorders (Table 1).

**Table 1.** Organelle-associated RBP/RNP components and their links to neurological diseases.

Organelle/platform	RBP/RNP components	Tethering/adaptor mechanism	Associated mRNA cohorts	Linked neurological diseases	Proposed pathogenic mechanism	References
**Lysosomes**	ANXA11	Phase separation (LCD) + Ca²+-dependent lysosomal membrane binding	Axonal maintenance and synaptic mRNAs	ALS	Impaired lysosomal hitchhiking → reduced distal mRNA delivery → axonal degeneration	Liao et al. (2019b), Marchica et al. (2025), Vargas et al. (2022)
**Late endosomes**	RAB7A-associated RNPs	Rab7-dependent pausing at mitochondria	Nuclear-encoded mitochondrial transcripts	CMT2B	Altered endosome motility → reduced endosome-associated translation → mitochondrial dysfunction	Cioni et al. (2019), Millecamps and Julien (2013), Vargas et al. (2022)
**Early endosomes (FERRY complex)**	FERRY complex (Rab5 effector)	Direct mRNA binding + Rab5-mediated recruitment	Mitochondrial and metabolic mRNAs	Neurodevelopmental and neurodegenerative phenotypes	Impaired mRNA capture into the endosomal RNA logistics pipeline	Schuhmacher et al. (2023), Quentin et al. (2023), Bauer and Koppers (2025)
**Axonal ER tubules**	p180/RRBP1-associated ribosomes	Ribosome tethering to ER membranes	Membrane and secretory protein mRNAs	Synaptic development defects	Loss of ER translation microdomains → impaired axon growth and synapse assembly	Koppers et al. (2024), Wu et al. (2017), Yperman and Kuijpers (2023)
**Mitochondria (outer membrane)**	SYNJ2BP–SYNJ2	mRNA anchoring to the mitochondrial surface	Pink1 and mitochondrial QC mRNAs	PD	Reduced mitochondria-localized translation → impaired mitophagy	Harbauer et al. (2022), Qin et al. (2021), Vargas et al. (2022)
**MERCs (Mitochondria–ER contacts)**	PERK-associated ISR components	ER–mitochondria contact-site signaling	Stress-adaptive and mitochondrial support mRNAs	AD, PD, ALS, FTD	Reduced spatial flexibility of translation under stress	Paillusson et al. (2016), Costa-Mattioli and Walter (2020), Hetz and Saxena (2017)
**RNP condensates (General)**	TDP-43, FUS, hnRNPA1	Aberrant phase separation + defective organelle docking	Synaptic and proteostasis mRNAs	ALS, FTD	Condensate dysregulation → mislocalized or stalled organelle-coupled translation	Banani et al. (2017), Vargas et al. (2022), Bauer and Koppers (2025)

### Disrupted lysosomal hitchhiking and ALS

ANXA11‑mediated lysosomal hitchhiking provides a direct physical link between RNP granules and motile lysosome‑related organelles, enabling long‑range co‑transport of mRNAs into axons where they can be locally translated to support synaptic and axonal maintenance (Liao et al., 2019; Vargas et al., 2022). This mechanism exemplifies how regulated tethering couples RNA logistics to organelle motility within the organelle‑anchored translation network (Liao et al., 2019; Vargas et al., 2022).

ALS‑associated ANXA11 mutations, many clustering in the low‑complexity N terminus or annexin repeat core, weaken condensate formation, lysosome binding, or both, causing RNP granules and lysosomes to mis‑segregate, stall, or abnormally cluster in neurites (Liao et al., 2019). Mechanistically, these mutations impair the multivalent interactions needed for stable condensate formation and membrane tethering, uncoupling mRNA transport from organelle dynamics (Liao et al., 2019).

In cellular and neuronal models, these defects correlate with impaired distal mRNA delivery, reduced local translation, and increased axonal degeneration, suggesting that failed lysosomal hitchhiking is a proximal mechanism by which ANXA11 mutations compromise axonal proteostasis and contribute to motor neuron vulnerability in ALS (Bauer and Koppers, 2025; Liao et al., 2019). More broadly, this phenotype illustrates that selective disruption of a single organelle–RBP interface can trigger compartment‑specific translational failure without globally disturbing RNA granule formation, directly linking molecular tethering defects to neurodegenerative pathology (Bauer and Koppers, 2025).

### Endosomal translation defects in Charcot–Marie–Tooth disease

Rab7‑positive late endosomes act as axonal translation platforms that pause at mitochondria and locally synthesize nuclear‑encoded mitochondrial proteins needed to maintain mitochondrial function and axonal integrity (Cioni et al., 2019; Vargas et al., 2022). This spatial coupling allows endosomal trafficking dynamics to control when and where mitochondrial support proteins are produced within long axons (Cioni et al., 2019).

In Charcot–Marie–Tooth disease type 2B (CMT2B), gain‑of‑function RAB7A mutations alter late endosome motility, maturation, and cargo handling, leading to abnormal endosome–mitochondria interactions and reduced local translation of mitochondrial support mRNAs in distal axons (Cioni et al., 2019; Millecamps and Julien, 2013). Rather than merely disturbing endocytic transport, these mutations selectively impair the ability of late endosomes to function as translation platforms, uncoupling organelle positioning from translational activation (Cioni et al., 2019).

These defects are associated with impaired mitochondrial homeostasis, increased axonal degeneration, and distal sensory loss, suggesting that disrupted endosome‑based translation forms a key mechanistic bridge between endocytic trafficking abnormalities and length‑dependent neuropathy in CMT2B (Bauer and Koppers, 2025; Millecamps and Julien, 2013). Together, these findings reinforce the idea that disease can arise from failure of organelle‑specific translational control even when global RNA granule dynamics remain largely intact (Bauer and Koppers, 2025).

### MERC and mitochondrial dysregulation in broader neurodegeneration

Mitochondria–ER contact sites (MERCs) are increasingly recognized as convergence points for pathogenic pathways shared across Alzheimer’s disease, Parkinson’s disease, ALS, and frontotemporal dementia (Paillusson et al., 2016; Rowland and Voeltz, 2012). Changes in MERC architecture and signaling are linked to defects in lipid exchange, calcium homeostasis, mitochondrial bioenergetics, and stress signaling, all recurrently perturbed in vulnerable neurons (Hetz and Saxena, 2017; Paillusson et al., 2016). Rather than primary triggers, MERCs are best viewed as vulnerability nodes where disturbances in organelle dynamics, stress responses, and local proteostasis intersect.

Disruption of MERC integrity or MERC‑enriched signaling modules, such as PERK‑dependent branches of the integrated stress response, can worsen mismatches between global translational repression and compartment‑specific protein demands (Costa-Mattioli and Walter, 2020; Hetz and Saxena, 2017). In this setting, MERCs may accelerate disease progression by limiting neurons’ ability to locally adjust translational output in axons and synapses during chronic stress, even when bulk translational control remains largely intact (Hetz and Saxena, 2017).

MERC dysfunction is unlikely to act alone; it converges with defects in RNA‑binding proteins, organelle trafficking, and phase‑separated condensates that control mRNA localization and translation. Perturbations in mitochondrial dynamics or ER stress signaling can destabilize organelle‑anchored RBP assemblies, whereas defects in RBP‑mediated mRNA tethering may increase dependence on MERC‑based stress buffering.

This reciprocal vulnerability positions MERCs as integrative nodes where failures in organelle–RBP coupling, mitochondrial maintenance, and stress adaptation reinforce one another. The framework predicts that MERC alterations sensitize neurons not by abolishing local translation outright, but by reducing the spatial flexibility with which translation can be prioritized under stress (Costa-Mattioli and Walter, 2020; Hetz and Saxena, 2017; Paillusson et al., 2016).

Such a model helps explain why MERC remodeling appears across diverse disorders with distinct genetic origins, and why restoring MERC homeostasis or stress‑adaptive signaling can be neuroprotective without fully correcting upstream insults (Costa-Mattioli and Walter, 2020; Hetz and Saxena, 2017; Paillusson et al., 2016). In this view, MERCs emerge as convergence points that translate heterogeneous molecular defects into shared phenotypes of synaptic failure and axonal vulnerability.

## Open questions and future directions

### Decoding the “tethering code” of RBP–organelle specificity

A central unresolved question in neuronal local translation is how RNA-binding proteins (RBPs) selectively associate with distinct organelles to form membrane-anchored condensates. The diversity of observed pairings—ANXA11 with lysosomes, FERRY with early endosomes, p180/RRBP1 with axonal ER tubules, and SYNJ2BP with mitochondria—supports a combinatorial “tethering code” that links RBP biophysical properties to organelle identity (Hung et al., 2017; Koppers et al., 2024; Liao et al., 2019; Schuhmacher et al., 2023).

Modular RBP architecture appears to be one layer of this code. RBPs involved in organelle-anchored translation typically combine low-complexity regions that drive multivalent condensation with discrete domains that mediate membrane association or adaptor binding, allowing the same protein to act as both condensate scaffold and spatial tether. Because condensation and membrane anchoring can be tuned independently, RBPs can switch between transport-competent, storage, and translation-permissive states without altering RNA recognition.

Organelle-specific docking environments provide a second layer. Distinct organelles differ in lipid composition, small GTPase signaling, and resident adaptors—e.g., acidic phospholipids on lysosomes and mitochondria, Rab5–FERRY and Rab7 platforms along the endosomal pathway, and membrane anchors such as SYNJ2BP or p180/RRBP1 that directly engage RBPs or ribosomes—which create permissive docking landscapes that bias specific RBP–organelle pairings and embed spatial information into translational control.

A third layer arises from regulated tethering. Neuronal activity, calcium, kinase pathways, and stress responses modify RBPs or adaptors, changing condensate material properties, membrane affinity, or interaction networks and shifting assemblies between long-range transport, repression, and local activation modes (Bauer and Koppers, 2025; Holt et al., 2019; Vargas et al., 2022). This regulation enables dynamic reassignment of translational capacity without wholesale changes in mRNA localization.

Disease-linked mutations highlight failure modes of the tethering code. Many neurodegeneration-associated variants in RBPs cluster in regions controlling phase behavior or membrane interaction rather than RNA binding, selectively destabilizing organelle–RBP coupling and producing compartment-restricted translational defects while sparing global granule formation (Bauer and Koppers, 2025; Liao et al., 2019). Together, these principles suggest that RBP–organelle specificity reflects a multilayered tethering code integrating RBP modularity, organelle docking environments, and regulatory signaling, whose decoding will be essential for predicting how translational tasks are spatially distributed and fail in disease.

### Visualizing condensate–organelle dynamics *in vivo*

A key challenge is to dissect organelle‑anchored translation in neurons by separating physical tethering from active protein synthesis and functional impact. In line with a three‑tier evidence framework, current tools can be grouped into: (i) detection of RBP–organelle coupling, (ii) readouts of translation near organelles, and (iii) functional tests of compartment‑specific proteostasis.

Tier 1 visualizes physical coupling between RBPs, mRNA‑rich condensates, and organelle membranes using live‑cell, super‑resolution, or correlative light–electron microscopy. Perturbing tethers such as ANXA11 or FERRY, which selectively disrupt colocalization and long‑range RNA transport, defines these assemblies as organized RNA logistics units rather than stochastic encounters, but does not yet prove active translation.

Tier 2 detects translation‑associated activity at organelle surfaces using approaches such as ribopuromycylation, puromycin labeling, proximity tagging of ribosomal proteins, or imaging of tagged ribosomal subunits. These assays show ribosome enrichment and nascent peptide synthesis near Rab7‑positive endosomes and p180/RRBP1‑decorated axonal ER, but typically average over transient pauses or bursts and thus offer limited temporal resolution and quantitative insight into sustained output.

Tier 3 provides functional validation that organelle‑anchored translation is required for compartment health. Disrupting Rab7 regulators or p180/RRBP1‑mediated ribosome anchoring causes compartment‑specific translational deficits together with defective axonal integrity, synapse formation, or mitochondrial maintenance, while leaving bulk translation and global RNA granule behavior relatively unchanged.

Bridging these tiers will require methods that track defined mRNA cohorts from transport through translation to functional consequences *in vivo*. Genetically encoded translation reporters, proximity‑restricted ribosome profiling, *in vivo* metabolic labeling, and advanced imaging such as lattice light‑sheet or MINFLUX microscopy promise to jointly resolve tethering, local translation, and physiological impact within intact neural circuits.

### Testable predictions emerging from the organelle-anchored translation framework

This framework yields several predictions that diverge from classical RNP-centric or purely global translational models.

First, disrupting specific organelle–RBP tethers should cause compartment-restricted translational defects without broadly altering RNA granule dynamics. Perturbing ANXA11–lysosome coupling or Rab7-dependent endosomal pausing, e.g., is expected to selectively reduce translation of axonal and mitochondrial support mRNAs in distal neurites while sparing somatic translation, testable with compartment-resolved ribopuromycylation, proximity-restricted ribosome profiling, or spatial metabolic labeling.

Second, disease-linked mutations that affect organelle dynamics or contact site architecture should preferentially impair translation of mRNAs whose localization depends on those organelles, rather than inducing uniform repression. ALS- or CMT-associated lesions are thus predicted to create organelle-signature translational fingerprints—such as selective loss of mitochondria-associated translation in long axons—detectable only by subcellular nascent proteome profiling, not bulk omics.

Third, MERC dysfunction should sensitize neurons to stress by reducing the spatial flexibility of translational prioritization rather than abolishing local translation. Under chronic proteotoxic or metabolic stress, neurons with compromised MERCs are predicted to show delayed or incomplete recovery of translation in mitochondria-proximal or synaptic compartments despite similar levels of global repression, a scenario testable with targeted live translation reporters.

Finally, restoring or rewiring organelle–RBP tethering should rescue local translation and neuronal resilience even in the presence of upstream disease insults. Strengthening RBP–organelle interactions, redirecting RBPs to alternative organelles, or stabilizing contact sites is predicted to normalize compartment-specific translation and slow degeneration without globally boosting protein synthesis, supporting the view that organelle-anchored translation factories are causal control points rather than passive correlates.

Together, these predictions frame neurodegeneration as a failure of spatial allocation of translational resources rather than a simple loss of overall translational capacity, and they outline concrete experimental routes to test the organelle-anchored translation model *in vivo*.

## Conclusion

Neuronal local protein synthesis can be understood as a distributed network of organelle-anchored translation factories, where RBP condensates are physically and functionally coupled to endolysosomal compartments, axonal ER tubules, mitochondria, and their contact sites. This framework moves beyond classical models of autonomous RNP granules or diffuse cytosolic translation by emphasizing how organelle identity, motility, and stress-responsive signaling impose spatial rules on when and where specific mRNAs are decoded.

Organizing recent findings into long-range transport modules, localized translation hubs, and stress-adaptive buffers highlights shared design principles—multivalency, phase separation, and regulated tethering—that together explain how neurons achieve rapid yet compartment-specific proteome remodeling across extended processes. Disruption of particular organelle–RBP interfaces, therefore yields selective vulnerabilities rather than global translational collapse, suggesting that neurodegenerative phenotypes reflect failures in the spatial allocation of translational resources more than simple loss of overall translation capacity.

Although most mechanistic evidence to date derives from neurons, these principles are likely to extend to other highly polarized cells, providing a general blueprint for organelle-anchored local translation. The predictions outlined here—compartment-specific translational defects, organelle-signature nascent proteomes, and stress-dependent loss of translational flexibility—offer concrete and testable criteria for evaluating this framework *in vivo*. Viewed from this perspective, organelle-anchored translation factories emerge as a unifying organizational principle of synaptic proteostasis and as experimentally tractable control points for dissecting—and ultimately stabilizing—local translation in disease-vulnerable neural circuits.

## Abbreviations

ALS: amyotrophic lateral sclerosis
AMPK: AMP-activated protein kinase
ANXA11: annexin A11
ATP: adenosine triphosphate
CMT: Charcot–Marie–Tooth disease
CMT2B: Charcot–Marie–Tooth disease type 2B
ER: endoplasmic reticulum
eIF2: eukaryotic translation initiation factor 2
FERRY: five-subunit endosomal Rab5 and RNA/ribosome intermediary (FERRY) complex
FTD: frontotemporal dementia
GABARAP: GABA(A) receptor-associated protein
ISR: integrated stress response
LC3: microtubule-associated protein 1 light chain 3
MAM: mitochondria-associated membrane
MERCs: mitochondria–ER contact sites
mRNP: messenger ribonucleoprotein
p180: ribosome receptor p180 (RRBP1)
PERK: PKR-like ER kinase (EIF2AK3)
PINK1: PTEN-induced kinase 1
Rab5: Ras-related protein Rab5
Rab7: Ras-related protein Rab7
RBP: RNA-binding protein
RNP: ribonucleoprotein
RRBP1: ribosome-binding protein 1 (p180)
SYNJ2: synaptojanin 2
SYNJ2BP: synaptojanin 2 binding protein
UPR: unfolded protein response.
